# Zona Incerta GABAergic Output Controls a Signaled Locomotor Action in the Midbrain Tegmentum

**DOI:** 10.1523/ENEURO.0390-19.2020

**Published:** 2020-02-14

**Authors:** Sebastian Hormigo, Ji Zhou, Manuel A. Castro-Alamancos

**Affiliations:** Department of Neurobiology and Anatomy, Drexel University College of Medicine, Philadelphia, PA 19129

## Abstract

The zona incerta is a subthalamic nucleus proposed to link sensory stimuli with motor responses to guide behavior, but its functional role is not well established. Using mice of either sex, we studied the effect of manipulating zona incerta GABAergic cells on the expression of a signaled locomotor action, known as signaled active avoidance. We found that modulation of GABAergic zona incerta cells, but not of cells in the adjacent thalamic reticular nucleus (NRT), fully controls the expression of signaled active avoidance responses. Inhibition of zona incerta GABAergic cells drives active avoidance responses, while excitation of these cells blocks signaled active avoidance mainly by inhibiting cells in the midbrain pedunculopontine tegmental nucleus (PPT). The zona incerta regulates signaled locomotion in the midbrain.

## Significance Statement

The zona incerta is an enigmatic nucleus in the forebrain whose functional role is not well established. We found that GABAergic cells in the zona incerta, that project to the midbrain, control the ability of mice to avoid a threat signaled by a sensory stimulus. Inhibiting these cells drives avoidance responses, while exciting them blocks avoidance responses by inhibiting targets in the midbrain.

## Introduction

In accordance with its extensive efferent and afferent projections, the zona incerta has been implicated in diverse behaviors (Mitrofanis, 2005). Zona incerta GABAergic projections to the posterior (PO) thalamus regulate thalamocortical transmission (Trageser and Keller, 2004; Lavallée et al., 2005; Trageser et al., 2006), projections to the paraventricular thalamus have a role in binge eating (Zhang and van den Pol, 2017), and projections to the periaqueductal gray are involved in defensive behaviors (Chou et al., 2018). Also, parvalbumin-positive neurons in the zona incerta that receive projections from the central amygdala have been implicated in Pavlovian fear conditioning (Zhou et al., 2018). Furthermore, zona incerta GABAergic neurons can control predatory hunting (Zhao et al., 2019). Considering the putative role of zona incerta in thalamocortical transmission, defensive behaviors and fear, we investigated its possible role in the expression (performance) of signaled active avoidance.

During signaled active avoidance, animals avoid a harmful footshock unconditioned stimulus (US) by moving between compartments in a cage (shuttling) during an interval signaled by a conditioned stimulus (CS; Mowrer, 1960; Bolles, 1970; Mineka, 1979; LeDoux et al., 2017). Active avoidance is a learned locomotor action supported by negative reinforcement; the harmful outcome is contingent on the behavior of the animal, if the animal shuttles during the CS presentation, the US does not occur. Avoidance is part of daily human behavior, such as exiting a building during an alarm (active avoidance) or crossing the street in response to the appropriate light (passive and active avoidance). Performance of active avoidance likely involves the interplay between sensory neural circuits that process the CS (auditory, somatosensory, or visual modality) and motor circuits that drive the conditioned locomotor response, through intermediate circuits that gate responding to the CS. The substantia nigra pars reticulata (SNr), a main output of the basal ganglia, appears to have such a gating role because it fully controls signaled active avoidance (Hormigo et al., 2016); excitation of SNr GABAergic cells blocks signaled active avoidance while inhibition drives active avoidance. The control exerted by SNr occurs in the midbrain pedunculopontine tegmental nucleus (PPT), which is part of the midbrain locomotor region (MLR; Shik et al., 1966; Skinner and Garcia-Rill, 1984; Ryczko and Dubuc, 2013; Mena-Segovia and Bolam, 2017) and an essential junction for the expression of signaled active avoidance (Hormigo et al., 2019). However, it is unlikely that the control exerted by SNr in PPT is unique. Other brain areas may similarly influence active avoidance in PPT depending on behavioral contingencies. The zona incerta is a good candidate because it has been generally proposed to have a gating role between sensory stimuli and motor responses (Mitrofanis, 2005), and because of the already mentioned putative roles in thalamocortical transmission, defensive behaviors and fear. Moreover, zona incerta cells project to PPT (Kolmac et al., 1998; Mitrofanis, 2005), which has an essential role in active avoidance (Hormigo et al., 2019), and to other sites also innervated by SNr cells, such as superior colliculus in the midbrain, and separate portions of the thalamus (Di Chiara et al., 1979; Barthó et al., 2002). Like SNr GABAergic cells, zona incerta GABAergic cells may be able to control signaled active avoidance.

We used cell type-specific optogenetic methods that systematically test different optogenetic patterns, by adjusting the light frequency and intensity, in a repeated measures design where animals serve as their own controls. Thus, animals perform signaled active avoidance (control) trials and some of these trials randomly occur with optogenetic stimulation of different patterns. Additional experiments also tested the possibility that the light alone had non-specific effects. We explored the putative role of zona incerta GABAergic cells in the expression of signaled active avoidance and found that zona incerta GABAergic cells mimic the effects of SNr GABAergic cells. Inhibition of zona incerta GABAergic cells promotes active avoidance responses. Conversely, excitation of zona incerta GABAergic cells strongly suppresses signaled active avoidance through projections to PPT in the midbrain. Zona incerta and SNr provide independent GABAergic channels for regulating active avoidance responses in the midbrain tegmentum.

## Materials and Methods

### Experimental design and statistics

All procedures were reviewed and approved by the Animal Care Committee of Drexel University and conducted in adult (more than eight weeks) male and female mice. The results from both sexes were combined since there is no sex difference in the behavior measured for the strains used (Hormigo et al., 2019).

All experiments involved a repeated measures design in which the mice or cells serve as their own controls. All conclusions derive from within-subjects comparisons; independent comparisons between different groups of mice or cells were not performed. We tested for a main effect (light) using a two-way mixed design ANOVA followed by comparisons with Tukey’s test. In the mixed design ANOVA, the repeated measures factor was the light effect (with as many levels as conditions tested) and the other factor was the animals’ sessions (behavior) or the repetition of the same protocols (cells). The sessions factor is statistically independent because the fully trained animals must perform the control signaled active avoidance trials (ACS trials) at the same high levels per session (by definition one session has no effect on task performance on the other sessions). Tukey’s tests were conducted for the repeated measures factor when the within-subjects effect (*F* value) was statistically significant at a level of *p* < 0.01. Behavioral optogenetic experiments consist of a balanced design wherein the tested light and control conditions (trials) are randomly distributed within the same session, which is repeated on multiple days. Thus, all comparisons are between conditions presented within the same session. We highlight results with significance levels of at least 0.01. To test for equivalency between ACS and LCS alone trials, we first used the mixed ANOVA Tukey’s to establish that the trials were not significantly different (*p* > 0.01). This was followed by an equivalence paired test in which the upper boundary for equivalence was set at 20%.

Power analysis was conducted with OriginLab Pro using the measured means difference variability. It revealed that three animals in which we conducted five identical daily sessions per animal (15 sessions) was sufficient to detect a ∼20% change in avoidance rate with a power of 0.99 (*p* < 0.05). This was the bare minimum number of animals and sessions per group.

The experiment timeline included the following sequence of phases: AAV injections (unless mice natively expressed opsins), active avoidance learning phase, optical fiber implantation, active avoidance testing phase, and in vivo/vitro recordings (some cases) followed by histology. In mice subjected to AAV injections (optogenetics), the active avoidance testing phase commenced three weeks after AAV injections. In mice subjected to optogenetics, optical fiber implantation occurred 5–6 d prior to commencing the active avoidance testing phase, which lasted three to five weeks with daily (weekday) sessions.

In order to enable rigorous approaches, we maintain a local server with a central database accessed through a wiki that logs all details and metadata related to the experiments, including all information about animals and details about surgical procedures, behavioral sessions, electrophysiological recordings, histology and scripts used for analyses. Moreover, during daily behavioral sessions or electrophysiological recordings, computers run experiments automatically using preset parameters logged for reference during analysis. Analyses are performed using scripts that automate all aspects of data analysis from access to metadata and data files to population statistics and graph generation (scripts and metadata will be accessible through our website or by request).

### Strains and AAVs

As noted in the results, the following AAVs (injected undiluted) and mouse strains were used in the present study. To inhibit zona incerta GABAergic cells in Vgat-cre mice (Jax 028862), we used AAV5-EF1a-DIO-eArch3.0-EYFP (UNC Vector Core, titers: 3.4 × 10^12^ GC/ml by Dot Blot). To excite zona incerta GABAergic cells in Vgat-cre mice, we used AAV5-EF1a-DIO-hChR2(H134R)-eYFP (UPenn Vector Core, titers: 1.8 × 10^13^ GC/ml by quantitative PCR). To excite CaMKIIa-expressing zona incerta cells in C57BL/6J mice (Jax 000664), we used AAV5-CaMKIIa-hChR2(H134R)-EYFP (UNC Vector Core, titers: 6.2 × 10^12^ GC/ml by Dot Blot). To inhibit PO thalamus cells in C57BL/6J mice, we used AAV5-CaMKIIa-eArchT3.0-EYFP (UNC Vector Core, titers: 4 × 10^12^ virus GC/ml by Dot Blot). As a no-opsin AAV control, we used AAV8-hSyn-EGFP (Addgene, titers: 4.3 × 10^12^ GC/ml by quantitative PCR).

### Surgeries

Optogenetics experiments involved bilaterally injecting a volume of 0.3-μl AAVs per site during isoflurane anesthesia (∼1%). Animals received carprofen after surgery. The stereotaxic coordinates for injection sites (in mm from bregma; lateral from the midline; ventral from the bregma-λ plane) are: zona incerta (2.3 posterior; 1.7; 3.7), thalamic reticular nucleus (NRT; 0.9–1.35 posterior; 1.8–2; 3.1).

In optogenetics experiments, a dual optical fiber (200 μm in diameter) was implanted bilaterally during isoflurane anesthesia at the above-mentioned coordinates and held in place with a combination of screws, cyanoacrylate and dental cement. Bilateral optical fibers were implanted in the injection site or in the projection site of the AAV injection site. The stereotaxic coordinates for the implanted optical fibers (in mm) are: zona incerta (2.3 posterior; 1.5; 3.8), NRT (0.9–1.35 posterior; 2; 3.1), PO thalamus (2.3 posterior; 1.5; 2.6), superior colliculus (4 posterior; 1–1.5; 1.5–1.8), and PPT (4.7 posterior; 1.25; 3.1 entering in the posterior direction at a 20° angle). The coordinate ranges reflect different animals that were combined together because the slight coordinate differences produced similar effects.

### Active avoidance

Mice were trained in the active avoidance task using procedures similar to those described previously for rats and mice (Cohen and Castro-Alamancos, 2007, 2010b; Hormigo et al., 2016). Mice were trained prior to reaching the testing phase; learning occurred during four to six daily sessions (50 trials per session) after AAV injections and prior to optical fiber implantation (see timeline above). All reported active avoidance data were gathered during the testing phase.

During an active avoidance session, mice are placed in a standard shuttle box (16.1″ × 6.5″) that has two compartments separated by a partition with side walls forming a doorway that the animal has to traverse to shuttle between compartments. For active avoidance training during the learning phase, a trial consists of a 7-s avoidance interval followed by a 10-s escape interval. During the avoidance interval, an auditory CS (ACS; 8 kHz, ∼85-dB tone) is presented for the duration of the interval or until the animal produces a conditioned response (avoidance response) by moving to the adjacent compartment, whichever occurs first. If the animal avoids, the CS ends, the escape interval is not presented and the trial terminates. However, if the animal does not avoid, the escape interval ensues consisting of white noise plus a mild scrambled electric footshock (0.3 mA) delivered through the grid floor of the occupied half of the shuttle box. This US readily drives the animal to move to the adjacent compartment (escape response), at which point the US terminates, ending the escape interval and the trial. Each trial is followed by an intertrial interval (duration is randomly distributed; 25- to 45-s range) during which the animal awaits the next trial and is free to cross between compartments (intertrial crossings). The main variables representing task performance are the percentage of avoidances (avoids) and the response latency from CS onset (time at which the animal enters the safe compartment).

### Optogenetics

The implanted dual optical fibers were connected to patch cables using sleeves. A black aluminum cap covered the head implant and completely blocked any light exiting at the ferrules junction. Furthermore, the experiments occurred in a brightly lit cage that makes it difficult to detect any light. The other end of the patch cables was connected to a dual light swivel (Doric lenses) that was coupled to a blue laser (450 nm; 80 mW) or a green laser (520 nm; 100 mW).

The blue light stimuli used during optogenetics included continuous pulses and trains of 1-ms pulses at 2, 5, 10, 20, 40, 66, 100 Hz. Three different blue light power levels were tested in different sessions (low, 0.5–1 mW; medium, 1.5–2.5 mW; high, 5.5–6.5 mW); at least two power levels were tested per group in different sessions. Sessions using medium blue light were conducted in all animals. If the medium power produced weak or nil effects on active avoidance, the High power was also tested. Conversely, if the medium power produced strong effects on active avoidance, the low power was also tested. The green light used to activate Arch was always continuous (Cont) and was tested at different powers (3–45 mW) randomly delivered within the session. Power is regularly measured by flashing the connecting patch cords onto a light sensor, with the sleeve on the ferrule.

The electrophysiological effects of these optogenetic light patterns (Cont and 2- to 100-Hz trains of 1-ms pulses) were previously characterized using both whole-cell recordings in slices and single-unit recordings in anesthetized mice (Hormigo et al., 2016, 2019). Similar validation experiments in some of the animals from this study verified the conclusions obtained previously. Briefly, continuous green light robustly inhibits cells that express eArch3.0 or eArchT3.0 as a function of light intensity. Regarding blue light applied to the soma-dendritic regions of ChR2-expressing GABAergic cells, trains of pulses (1 ms) increase cell-firing rates as a function of both frequency and intensity, plateauing at around 40–66 Hz. For the same light intensity, continuous pulses evoke the strongest cell firing, so that there is an increase in cell firing rate as a function of train frequency that is maximal during continuous light (Hormigo et al., 2016). Regarding blue light applied to fibers or synaptic terminals of ChR2-expressing GABAergic cells at their targets, trains of 1-ms pulses at 40- to 66-Hz drive the most sustained IPSPs in postsynaptic neurons. In contrast, continuous pulses drive strong IPSPs at light onset but the IPSPs rapidly adapt (Hormigo et al., 2019). Thus, 40- to 66-Hz blue light trains drive the strongest sustained inhibition, while continuous pulses of blue light drive strong inhibition only at light onset. Since this spectrum of excitation and inhibition patterns can occur in brain circuits under different conditions, it is important to consistently test (during behavior) the effects of continuous pulses and different frequency trains of light at varying intensities, as done in the present study.

### ACS trial types

**Figure 1.**
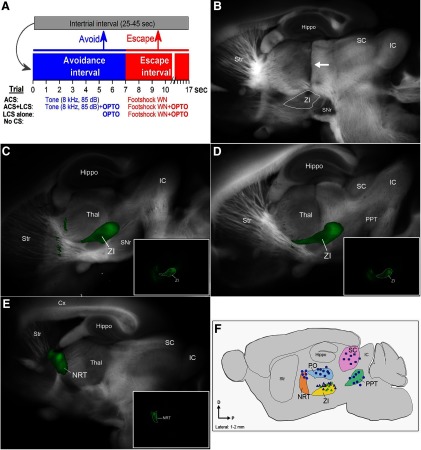
Active avoidance procedure and location of AAV injections and optical fibers. ***A***, Schematic of the active avoidance procedure showing different trial types. ***B***, Example of a parasagittal section showing a cannula tract (arrow) coursing to zona incerta. ***C***, ***D***, Example AAV injections in the zona incerta. The images blend a light image of the section with the green channel of the eYFP fluorescent image. The inset photomicrographs show the eYFP fluorescence alone. ***E***, Example AAV injections in NRT. The images blend a light image of the section with the green channel of the eYFP fluorescent image. The inset photomicrograph shows the eYFP fluorescence alone. ***F***, Reconstruction of optical fiber track endings in the zona incerta, PO thalamus, superior colliculus, and PPT for brains cut in the sagittal plane (1–2 mm lateral from the midline).

Within daily sessions, we tested different types of trials (Fig. 1*A*) presented randomly. In ACS trials, an ACS (8-kHz tone at ∼85 dB) signals the avoidance interval. ACS trials are control trials that represent the normal performance of the animal during a session. Every block of ten trials within an optogenetics session has five randomly presented ACS trials to assure that the animal continues to perform the task in the absence of the optogenetic manipulation. In ACS+LCS trials, the avoidance interval is signaled by the same ACS as in ACS trials and optogenetic light is simultaneously delivered into the brain bilaterally. The purpose of ACS+LCS sessions is to compare ACS+LCS trials versus ACS trials in order to determine whether the optogenetic manipulation has any effect on avoidance responses driven by the ACS. The blue light used to activate ChR2 is tested within the same session using 8 different patterns of light delivered randomly at the same power (low, medium, or high). The green light used to activate Arch was always Cont and was tested at different powers randomly delivered within a session. In LCS alone trials, the avoidance interval is signaled only by light delivered into the brain. The purpose of LCS alone sessions is to compare LCS alone trials versus ACS trials in order to determine whether the optogenetic manipulation is capable of driving avoidance responses as effectively as the ACS. Thus, in LCS alone sessions, we indicate non-significant differences (*p* > 0.01) between LCS alone trials and ACS trials to highlight optogenetics stimuli that were equivalent to the ACS in driving avoidance responses. In no-CS trials, the avoidance interval is not signaled by any stimulus and the escape interval does not occur. The purpose of no-CS trials is to determine the percentage of avoidance responses done by chance due to motor activity.

In optogenetics trials (ACS+LCS trials or LCS alone trials), the light persists during the escape interval but not during the intertrial interval. Thus, the optogenetic light delivered per trial depends on the duration of the avoidance (maximum 7 s) and escape intervals (<3 s for all mice), which the animals control. All optogenetics experiments involved a repeated measures design in which different randomly presented trials are compared within the same session (50–150 trials per session). Half of the trials in a session are control trials (e.g., ACS trials) and the other half are optogenetics trials (ACS+LCS or LCS alone trials) that employed different light stimuli. Statistical comparisons consist of repeated measures between different trial types tested within a session [significant statistical differences vs ACS trials (*p* < > 0.01) are indicated in each figure].

### Video tracking

Animals are video tracked (30 FPS) during active avoidance sessions that employed optogenetics. The tracking followed color markers located on the head connector above the nose and between the ears. Several movement (tracking) measures were derived during active avoidance. Distance was the number of pixels crossed by the animal in its trajectory during the avoidance and escape intervals of a trial (trial distance) or during the intertrial interval (intertrial distance). Displacement was the number of pixels in a straight line between the position of the animal at trial start and the position of the animal at trial end (when the animal avoided or escaped). Pixel measures were converted to cm using calibrations. Trial speed was the trial distance divided by the response latency. Intertrial speed was the intertrial distance divided by the intertrial interval duration. Trial velocity was the displacement divided by the response latency. Response onset was estimated by calculating the first derivative of the speed and determining the point in time at which it crossed a baseline threshold (mean ± SD of the pre-trial speed measured during 1 s before trial start) for at least 350 ms.

### Histology

Mice were deeply anesthetized with an overdose of ketamine and on losing all responsiveness to a strong tail pinch, the animal was decapitated and the brain was rapidly extracted and placed in fixative for histologic processing. The brain was sectioned (100-μm sections) in the coronal or sagittal planes. Sections were mounted on slides, cover-slipped with DAPI mounting media, and photographed using a fluorescent microscope. The location of the tips of the implanted optical fibers derived from histologic sections were marked on a standard atlas (Franklin and Paxinos, 2008), which is redrawn flattened in the parasagittal plane (Fig. 1).

## Results

### Inhibition of zona incerta GABAergic cells drives active avoidance responses

In signaled active avoidance, a trial consists of consecutive avoidance and escape intervals followed by a random intertrial interval (Fig. 1*A*). Depending on the trial type, a different CS is presented during (signals) the avoidance interval. In ACS trials, an auditory tone CS (ACS) signals the avoidance interval. In ACS+LCS trials, the same ACS used in ACS trials signals the avoidance interval and optogenetic light is delivered simultaneously into the brain during the avoidance interval. In LCS alone trials, the avoidance interval is signaled only by light delivered into the brain. In no-CS trials, the avoidance interval is not signaled by any stimulus and the escape interval does not occur.

To determine the effect of inhibiting zona incerta GABAergic cells on signaled active avoidance, Vgat-cre mice received bilateral injections of a Cre-inducible AAV (AAV5-EF1a-DIO-eArch3.0-EYFP; UNC Vector Core) in the zona incerta to express eArch3.0 in GABAergic cells (Vgat-ZI-Arch). A dual optical fiber was implanted in the zona incerta of these animals. Figure 1 shows typical AAV injections in zona incerta (Fig. 1*C*,*D*) and the location of the optical fibers track endings for optical fibers implanted in zona incerta (Fig. 1*B*). The green light used to activate Arch in optogenetics trials (ACS+LCS and LCS alone trials) was Cont and tested at different powers (3–45 mW) randomly delivered within the same session.

**Figure 2.**
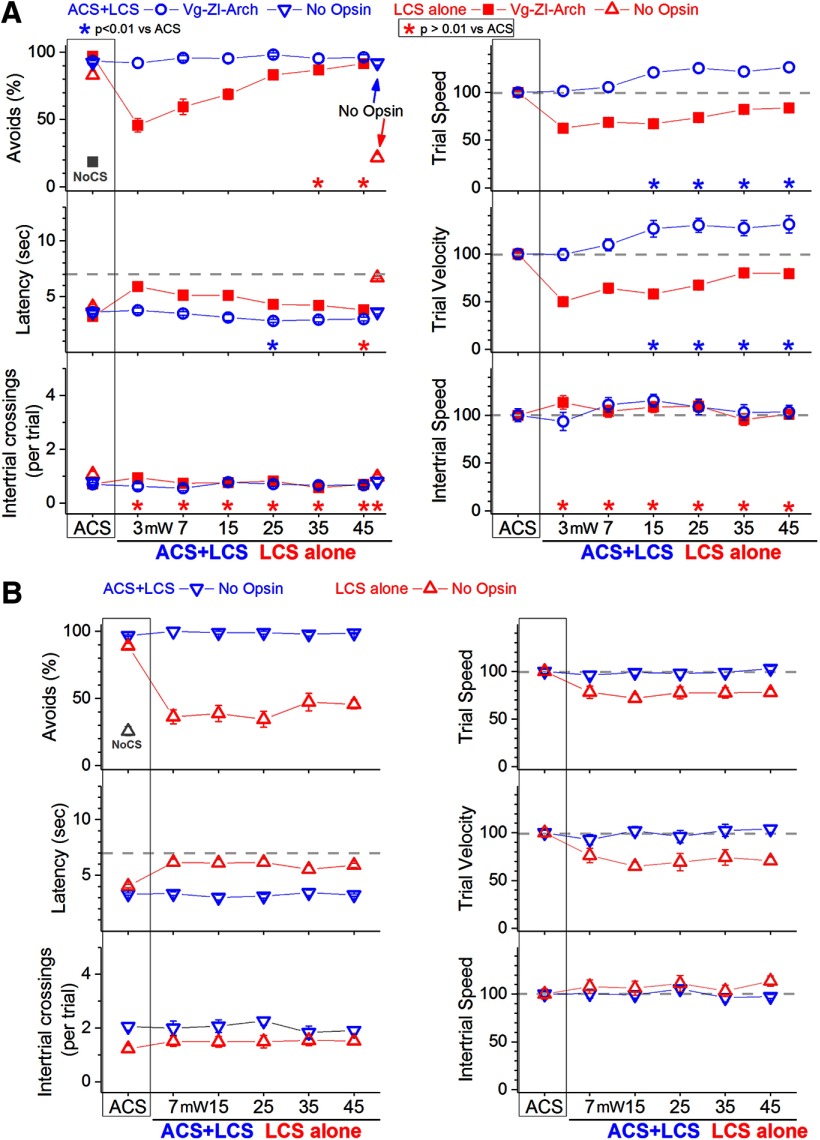
Effect of inhibiting zona incerta GABAergic cells on active avoidance responses. ***A***, Effect of green light applied in the zona incerta on ACS+LCS trials (blue) and on LCS alone trials (red) for mice that express eArch3.0 in GABAergic zona incerta cells (Vgat-ZI-Arch). ACS+LCS trials measure the effect of optogenetic stimulation on avoidance responses driven by the ACS. LCS alone trials measure the ability of the optogenetic stimulation to drive avoidance responses in the absence of the ACS. Plots in all figures display mean ± SEM, and asterisks denote Tukey’s tests. The plots also show data for the no opsin group of animals (open triangles), which compares the effect of all the light patterns used (combined together and delivered in various brain regions) versus ACS. The right panels show trial speed, trial velocity, and intertrial speed for the data in the left panels. The *x*-axis denotes green light power in mW. ***B***, Effect of green light applied specifically in the zona incerta at different light powers on ACS+LCS trials (blue) and on LCS alone trials (red) for no opsin mice. The green light applied in zona incerta without opsin activation does not affect avoidance responses driven by the ACS and is not able to effectively drive avoidance responses in the absence of the ACS. The *x*-axis denotes green light power in mW.

In ACS+LCS trials, inhibition of zona incerta GABAergic cells with different continuous green light powers (3–45 mW) did not change the percentage of avoidance responses compared with ACS trials (Fig. 2*A*, left panels, blue open circles, Vgat-ZI-Arch; 35 sessions in five mice; *F*_(6,162)_ = 33.1, *p* = 0.3). There was a small reduction in response latency but only for green light power at 25 mW (Tukey, *p* = 0.0052). Video tracking during the task showed (Fig. 2*A*, right panels) an increase in both trial speed (*p* < 0.00001, *p* < 0.00001, *p* < 0.00001, and *p* < 0.00001; 15, 25, 35, and 45 mW vs ACS trials) and trial velocity (*p* = 0.0068, *p* < 0.00001, *p* = 0.0058, and *p* = 0.0011; 15, 25, 35, and 45 mW vs ACS trials) compared with ACS trials for all green light powers above 7 mW. Thus, inhibition of zona incerta GABAergic cells increases the speed of avoidance responses.

In LCS alone trials, inhibition of zona incerta GABAergic cells (Fig. 2*A*, red closed squares, Vgat-ZI-Arch; 38 sessions in five mice) functioned as a very effective CS to drive avoidance responses in the absence of an external ACS. The percentage of avoidance responses in LCS alone trials was much higher than in no-CS trials and was not statistically different from ACS trials for the light powers above 25 mW (Tukey, *p* = 0.02 and *p* = 0.53; 35 and 45 mW vs ACS trials). Response onset was equivalent to ACS trials at the highest light power used (45 mW; *p* = 0.5; data not shown). However, response latency was still slightly slower compared with ACS trials at this power (45 mW; *p* = 0.009; Fig. 2*A*), suggesting that animals avoided with a slower speed. Indeed, trial speed and velocity (Fig. 2*A*) were significantly different from ACS trials for all green light intensities, although these differences were minor at the higher light powers. Thus, inhibition of zona incerta GABAergic cells is an effective CS to drive avoidance responses, and these responses are slightly slower than those evoked by the natural ACS.

During active avoidance, a black aluminum cap completely covered the head implant where the dual optical fiber and the optic fibers connected, blocking any exiting light. To ensure that light per se had no effect, we implanted Vgat-Cre mice that did not express opsins (because they were injected with AAV8-hSyn-EGFP, AddGene, or not injected with an AAV; no opsin mice) with bilateral optical fibers in various brain regions (zona incerta, PPT, and SNr; *n* = 8). In daily sessions, we presented ACS+LCS trials or LCS alone trials (continuous light) with high green or high blue light powers (35 and 6 mW, respectively; sessions were combined after determining that they had no effect when considered separately). In ACS+LCS trials (Fig. 2*A*, blue open triangles; 27 sessions in five mice), application of light to no opsin mice had no effect on the percentage of avoidance responses (Tukey, *p* = 0.9), response latency (*p* = 0.9), or the number of intertrial crossings (*p* = 0.94) compared with ACS trials. In LCS alone trials (Fig. 2*A*, red open triangles; 44 sessions in five mice), application of light to no opsin mice was not effective at driving avoidance responses; the percentage of avoidance responses was similar to no-CS trials. Figure 2*B* specifically shows data from no opsin mice implanted in zona incerta (*n* = 3) and subjected to both ACS+LCS and LCS alone sessions using the different green light powers employed in the study. There was no effect on avoidance responses driven by the ACS (ACS+LCS sessions; 20 sessions in three mice; *F*_(5,85)_ =1.5, *p* = 0.2) and the light was not capable of driving avoidance responses in the absence of an external ACS (LCS alone sessions; 22 sessions in three mice; *F*_(6,114)_ = 33.1, *p* < 0.00001; none of the five LCS alone stimuli were equivalent to the ACS, Tukey, *p* < 0.00001). The light delivered into the brain was not an effective CS in the absence of opsin activation. Therefore, it is the inhibition of zona incerta GABAergic cells that drives faster avoidance responses to the ACS, and can substitute for the ACS to drive avoidance responses.

### Inhibition of zona incerta GABAergic cells in naive mice

One possibility is that inhibition of zona incerta GABAergic cells drives active avoidance responses in the absence of a sensory CS because it induces locomotor activity, which translates into avoidance responses. If this is the case, inhibiting zona incerta GABAergic cells should drive shuttling (i.e., trial crossings) in naive animals that have not experienced the US (i.e., have not learned that the LCS or ACS predict the US). To test this possibility, Vgat-cre mice received bilateral injections of a Cre-inducible AAV (AAV5-EF1a-DIO-eArch3.0-EYFP; UNC Vector Core) in the zona incerta to express eArch3.0 in GABAergic cells (Vgat-ZI-Arch-NoUS). A dual optical fiber was implanted in the zona incerta of these animals.

**Figure 3.**
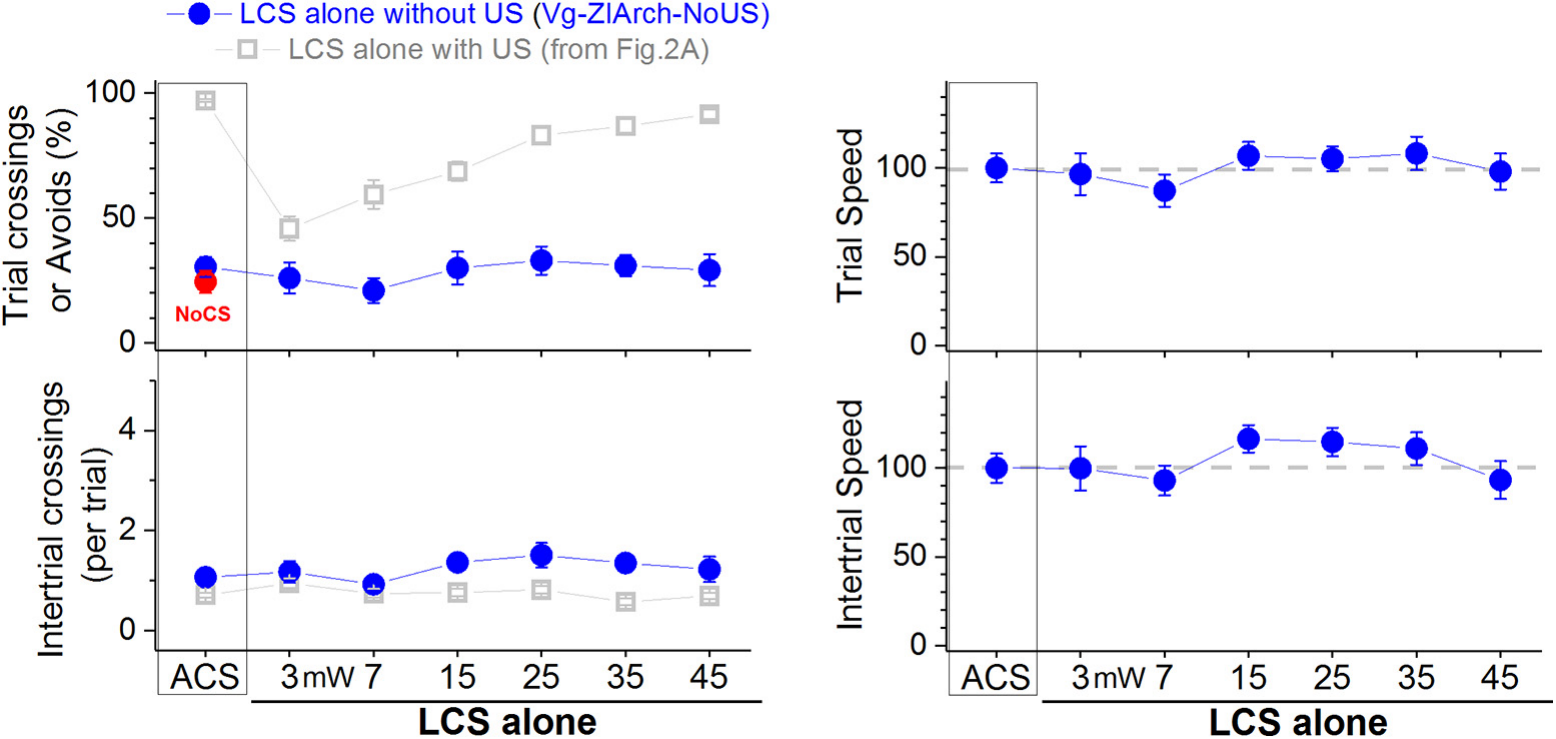
Effect of inhibiting zona incerta GABAergic cells in naive animals. Effect of green light applied in the zona incerta on LCS alone trials (without US) and ACS trials (without US) for mice that express eArch3.0 in GABAergic zona incerta cells (filled blue circles; Vgat-ZI-Arch-NoUs). The left panel displays trial and intertrial crossings (trial crossings are shuttling responses during the LCS or ACS, akin to avoids). The right panels display trial and intertrial speed for the data in the left panels. LCS alone trials without US measure the innate response of the optogenetic stimulation on shuttling and motor activity. For comparison, the left panels also overlay (open gray squares) data of LCS alone trials (with US) and ACS trials (with US) taken from Figure 2*A*. The *x*-axis denotes green light power in mW.

Mice were subjected to the same procedures as those shown in Figure 2*A*, red squares, except that the LCS alone and ACS trials did not include a US (without US); if the animals did not shuttle within 7 s of the LCS or ACS presentation, the intertrial interval started. Figure 3 shows trial crossings (equivalent to avoidance responses) and speed evoked by LCS alone trials and ACS trials without US (blue closed circles, Vgat-ZI-Arch-NoUS; 20 sessions in four mice). Mice did not shuttle in response to either the LCS alone or the ACS when these stimuli did not predict the US; the percentage of shuttling was not different compared with no-CS trials (*F*_(7,112)_ = 0.7, *p* = 0.6). Moreover, speed tracking during the sessions revealed that the optogenetic stimulation did not affect trial speed compared with presentation of the ACS or no-CS (*F*_(7,105)_ = 1.6, *p* = 0.13). Once the US was included (with US), animals learned to avoid the US in response to both types of stimuli (ACS and LCS alone). In conclusion, inhibition of zona incerta GABAergic cells in naive animals does not drive locomotor activity that can produce spurious trial crossings (avoidance responses). Inhibiting zona incerta GABAergic cells is equivalent to presenting a neutral sensory stimulus that like a true CS only begins to drive avoidance responses when it predicts the US.

### Excitation of zona incerta GABAergic cells blocks active avoidance responses

To determine the effect of exciting zona incerta GABAergic cells on signaled active avoidance, Vgat-cre mice were injected with a Cre-inducible AAV (AAV5-EF1a-DIO-hChR2(H134R)-eYFP; UPenn Vector Core) into the zona incerta to express ChR2 in GABAergic cells (Vgat-ZI-ChR2; Fig. 1*C*,*D*). These animals were implanted with a dual optical fiber in zona incerta (Fig. 1*B*,*F*). The blue light used during optogenetics trials to excite ChR2 included continuous pulses (Cont) and trains of 1-ms pulses (at 2, 5, 10, 20, 40, 66, and 100 Hz) randomly delivered within the session. Three different blue light power levels termed low (0.5–1 mW), medium (1.5–2.5 mW), and high (5.5–6.5 mW) were tested in different sessions; at least two power levels were tested per each group of animals (the medium power was tested in all animals).

**Figure 4.**
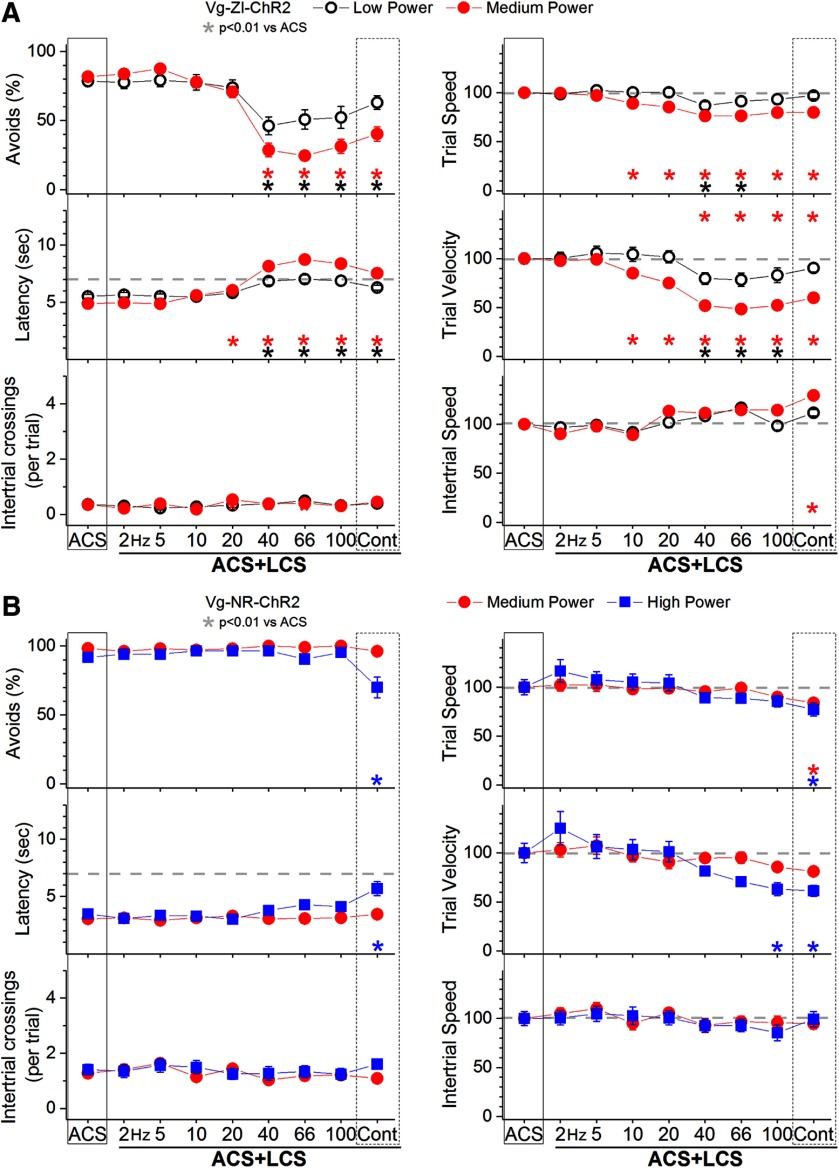
Effect exciting zona incerta GABAergic cells or NRT GABAergic cells on active avoidance responses. ***A***, Effect of low-power and medium-power blue light applied in the zona incerta on ACS+LCS trials for animals that express ChR2 in zona incerta GABAergic cells (Vgat-ZI-ChR2). The right panels show trial speed, trial velocity, and intertrial speed for the data in the left panels. The *x*-axis denotes blue light frequency trains of 1-ms pulses (Hz) or continuous pulses (Cont). ***B***, Effect of medium-power and high-power blue light applied in the NRT on ACS+LCS trials for animals that express ChR2 in NRT GABAergic cells (Vgat-NR-ChR2). The right panels show trial speed, trial velocity, and intertrial speed for the data in the left panels. The *x*-axis denotes blue light frequency trains of 1-ms pulses (Hz) or continuous pulses (Cont).

In ACS+LCS trials, excitation of zona incerta GABAergic cells with low or medium blue light powers strongly suppressed the percentage of avoidance responses compared with ACS trials (Fig. 4*A*, black open circles; 26 sessions in four mice). At low power, the suppression occurred for light trains above 20 Hz (Tukey, *p* < 0.00001, *p* < 0.00001, and *p* < 0.00001; 40, 66, and 100 Hz vs ACS) and continuous light (*p* = 0.005; Cont vs ACS). Response latency increased for light trains above 20 Hz (*p* < 0.00001, *p* < 0.00001, and *p* < 0.00001; 40, 66, and 100 Hz vs ACS) and continuous light (*p* = 0.008; Cont vs ACS). Trial speed decreased during trains at 40–66 Hz (*p* < 0.0001 and *p* = 0.003; 40 and 66 Hz vs ACS), while trial velocity decreased for trains at 40–100 Hz (*p* < 0.00001, *p* < 0.0001, and *p* = 0.005; 40, 66, and 100 Hz vs ACS). The number of intertrial crossings and intertrial speed were not affected. The medium light power replicated the results of the low power but the effects were stronger and occurred for a broader range of train frequencies (Fig. 4*A*, red closed circles; 37 sessions in four mice). When blue light trains suppressed avoidance responses, trial velocity (movement toward the safe compartment) was more strongly suppressed than trial speed (overall movement). The results show that excitation of zona incerta GABAergic cells strongly interferes with signaled active avoidance responses.

A group of no opsin mice implanted with optical cannulas in the zona incerta was tested on ACS+LCS sessions to determine whether the blue light at the highest powers used in the study could affect avoidance responses. We compared ACS trials and ACS+LCS trials consisting of continuous pulses and 40-Hz trains at the medium and high powers. Blue light in no opsin mice had no effect on the percentage of avoidance responses driven by the ACS (15 sessions in three mice; *F*_(4,48)_ = 0.49, *p* = 0.74). Thus, it is the excitation of GABAergic cells in zona incerta that suppresses signaled active avoidance responses, not the blue light per se.

### Excitation of NRT GABAergic cells does not block active avoidance responses

The NRT is a thalamic nucleus located proximal to zona incerta that also contains GABAergic neurons. Similar to zona incerta neurons, NRT neurons project to the thalamus (Jones, 1985). NRT neurons have been shown to have important roles in sensory processing during selective attention (McAlonan et al., 2006, 2008; Wimmer et al., 2015). Thus, it would be useful to determine whether excitation of NRT cells affect signaled active avoidance, like excitation of zona incerta cells does. To determine the effect of exciting NRT GABAergic cells on signaled active avoidance, Vgat-cre mice were injected with a Cre-inducible AAV [AAV5-EF1a-DIO-hChR2(H134R)-eYFP; UPenn Vector Core] into the NRT to express ChR2 in GABAergic cells (Vgat-NR-ChR2; Fig. 1*E*). These animals were implanted with a dual optical fiber in NRT (Fig. 1*F*).

In ACS+LCS trials, excitation of NRT GABAergic cells with medium or high blue light powers had nil or only modest effects on signaled active avoidance. At the medium power (Fig. 4*B*, red closed circles; 21 sessions in three mice; *F*_(8,112)_ = 1.7, *p* = 0.1 for avoids), which maximally blocks signaled active avoidance when applied in zona incerta, there was no effect on any parameter of the active avoidance task. Video tracking revealed a small (16%) suppression of trial speed only during continuous blue light (*p* = 0.004, Cont vs ACS). At the high power (Fig. 4*B*, blue closed squares; 17 sessions in three mice), there was a modest suppression of the percentage of avoidance responses only during continuous blue light (*p* < 0.00001; Cont vs ACS), which was associated with small changes in response latency (*p* < 0.00001), trial speed (*p* = 0.003) and trial velocity (*p* < 0.00001, Cont vs ACS; *p* = 0.01, 100 Hz vs ACS). The number of intertrial crossings were not affected.

These results highlight the selectivity of the zona incerta effects by showing that excitation of NRT GABAergic cells has nil or minor effects on signaled active avoidance compared with the strong suppression caused by same manipulation performed on zona incerta GABAergic cells. Moreover, since both zona incerta and NRT consist of GABAergic cells that have prominent projections to thalamus, but exciting NRT does not impair active avoidance, the effects of exciting zona incerta are unlikely caused by inhibition of targets in the thalamus, as shown below.

### Excitation of zona incerta GABAergic fibers in PO thalamus

The preceding results show that excitation of zona incerta GABAergic cells suppresses signaled active avoidance responses. Zona incerta cells principally project to the PO thalamus, superior colliculus and PPT (Barthó et al., 2002; Mitrofanis, 2005). Therefore, we stimulated zona incerta GABAergic fibers in these locations to determine whether these targets of zona incerta cause the suppression of avoidance responses. Vgat-cre mice were injected with a Cre-inducible AAV (AAV5-EF1a-DIO-hChR2(H134R)-eYFP; UPenn Vector Core) in the zona incerta (to express ChR2 in zona incerta GABAergic cells; Vgat-ZI-ChR2), and implanted with bilateral optical fibers in the PO thalamus, superior colliculus or PPT (Fig. 1*F*). Superior colliculus and PO thalamus optical fibers were inserted perpendicular to the bregma-λ plane. PPT optical fibers entered at a 20° angle in the posterior direction and targeted the dorsal and posterior portions of PPT. We first report the effects of exciting zona incerta GABAergic fibers in PO thalamus.

**Figure 5.**
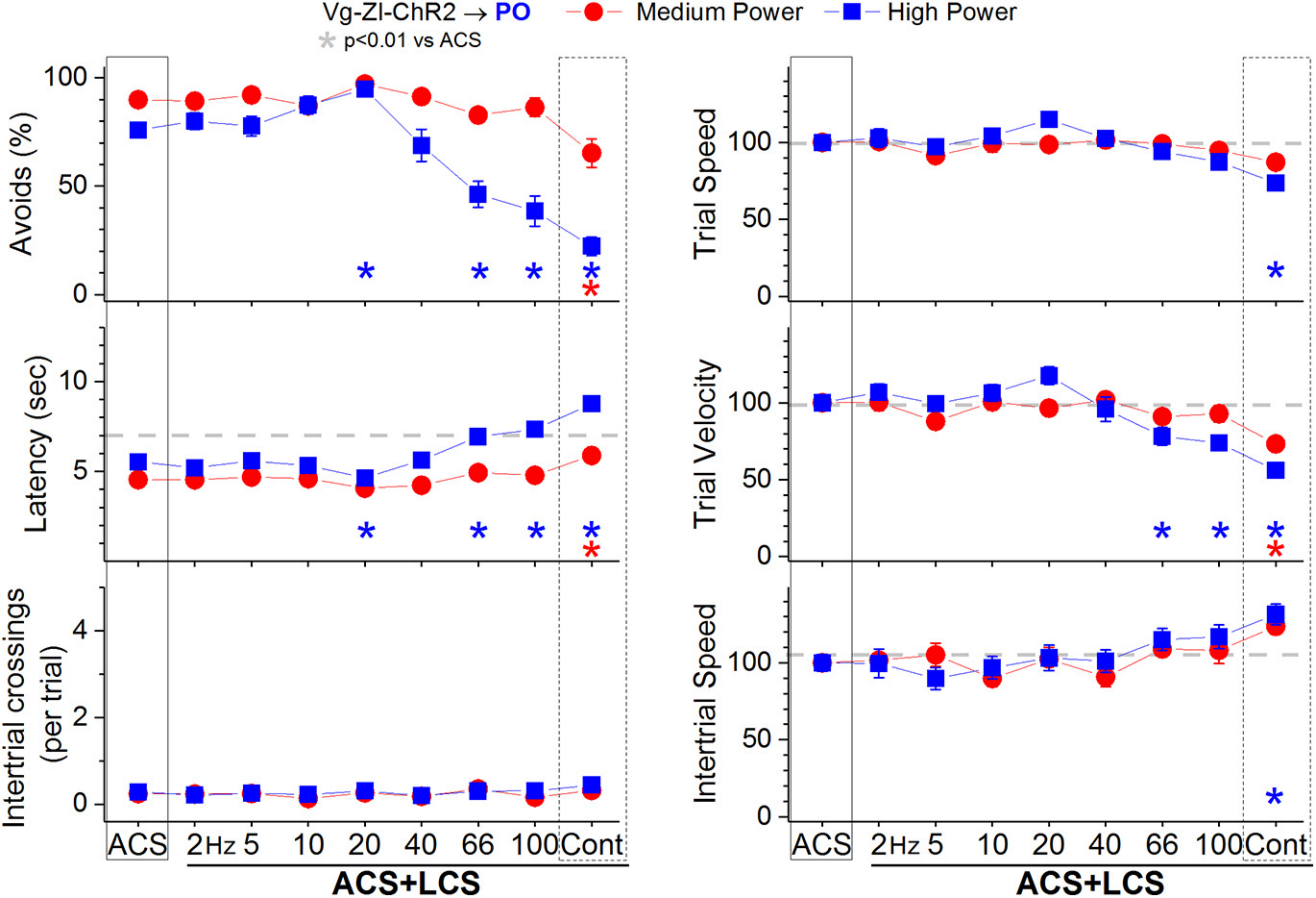
Effect of activating zona incerta GABAergic output fibers in PO thalamus on active avoidance responses. Effect of medium-power and high-power blue light applied in the PO thalamus on ACS+LCS trials for animals that express ChR2 in GABAergic fibers originating in the zona incerta (Vgat-ZI-ChR2→PO). The right panels show trial speed, trial velocity, and intertrial speed for the data in the left panels. The *x*-axis denotes blue light frequency trains of 1-ms pulses (Hz) or continuous pulses (Cont).

In ACS+LCS trials, excitation of zona incerta fibers in the PO thalamus (incertothalamic fibers) with medium blue light power (Fig. 5, red closed circles; 28 sessions in four mice) modestly suppressed the percentage of avoidance responses (*p* < 0.0001; Cont vs ACS), increased latency (*p* < 0.0001; Cont vs ACS), and suppressed trial velocity (*p* < 0.0001; Cont vs ACS), but only for continuous light. Increasing the blue light to high power (Fig. 5, blue closed squares; 27 sessions in four mice) resulted in a broader effect, so that trains above 40 Hz and continuous light suppressed the percentage of avoidance responses (*p* < 0.0001, *p* < 0.0001, and *p* < 0.0001; 66 and 100 Hz and Cont vs ACS), increased latency (*p* < 0.0001, *p* < 0.0001, and *p* < 0.0001; 66 and 100 Hz and Cont vs ACS), and suppressed trial velocity (*p* = 0.001, *p* < 0.0001, and *p* < 0.0001; 66 and 100 Hz and Cont vs ACS). Interestingly, high-power blue light trains at 20 Hz actually had the opposite effect; it slightly enhanced the percentage of avoidance responses (*p* = 0.001; 20 Hz vs ACS), decreased latency (*p* = 0.001; 20 Hz vs ACS), and increased trial velocity (*p* = 0.01; 20 Hz vs ACS). Thus, mainly the continuous high-power blue light robustly suppresses signaled active avoidance. However, when exciting GABAergic fibers, continuous pulses of blue light produce the weakest sustained inhibition in the postsynaptic cells due to strong IPSP adaptation compared with 40-Hz trains (Hormigo et al., 2019). This suggested that the observed suppression of active avoidance might not be caused by inhibiting PO thalamus cells, but may be due to the high-power blue light spreading to the zona incerta located below the PO thalamus. We address this possibility next.

### Inhibition of PO thalamus cells does not suppress signaled active avoidance

To determine whether inhibiting PO thalamus cells affects signaled active avoidance, we expressed eArchT3.0 in PO thalamus cells applying the same methods that were shown to block signaled active avoidance in PPT (Hormigo et al., 2019). Thus, to inhibit PO thalamus glutamatergic cells, an AAV with a CaMKII promoter (AAV5-CaMKIIa-eArchT3.0-EYFP; UNC Vector Core) was bilaterally injected in the PO thalamus of C57BL/6J mice (CaMKII-PO-Arch). These animals were implanted with a dual optical fiber in PO thalamus (Fig. 1*F*).

**Figure 6.**
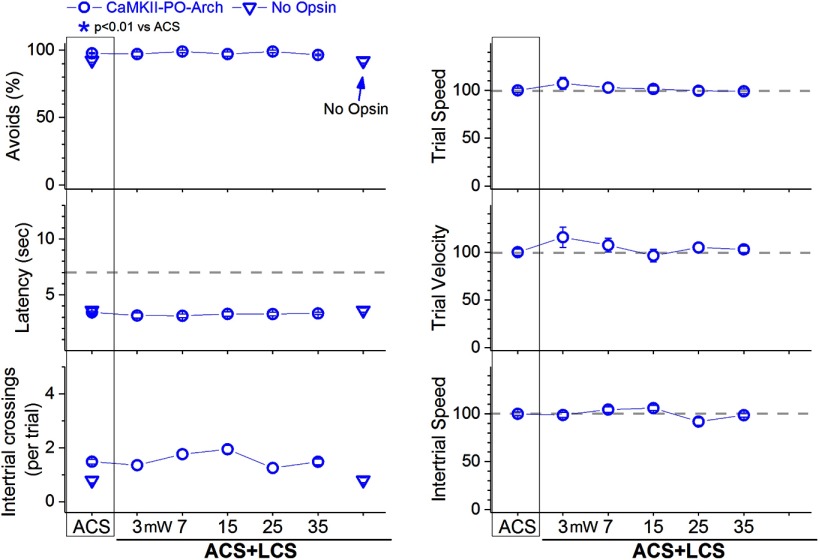
Effect of inhibiting PO thalamus cells on active avoidance responses. Effect of green light applied in PO thalamus on ACS+LCS trials (blue) for mice that express eArchT3.0 in glutamatergic PO thalamus cells (CaMKII-PO-Arch). The plots also show data for the no opsin group of animals (open triangles), which compares the effect of all the light patterns used (combined together and delivered in various brain regions) versus ACS. The right panels show trial speed, trial velocity, and intertrial speed for the data in the left panels. The *x*-axis denotes green light power in mW.

In ACS+LCS trials, inhibition of PO thalamus cells with different continuous green light powers (3–35 mW) did not change the percentage of avoidance responses compared with ACS trials (Fig. 6, left panels, blue open circles, CaMKII-PO-Arch; 25 sessions in five mice; *F*_(5,100)_ = 1.3, *p* = 0.24; 45 mW was also tested in a few sessions without effect). Video tracking during the task (Fig. 6, right panels) did not reveal significant effects on trial speed, trial velocity or intertrial speed. Inhibition of PO thalamus cells does not suppress signaled active avoidance. Therefore, the block of signaled active avoidance that occurs when continuous high-power blue light is applied in the PO thalamus of Vgat-ZI-ChR2 mice (Fig. 5, blue squares) is likely due to the light spreading to the underlying zona incerta, where it excites ChR2-expressing GABAergic cells that project to other areas different from PO thalamus.

We further tested this possibility (i.e., light spreading from PO thalamus to zona incerta located approximately 0.6–1 mm apart) by conducting electrophysiological experiments in vivo. First, we estimated the spread of blue light within the brain at the different powers used in our study. Assuming previously estimated values for blue light transmission through brain tissue (Aravanis et al., 2007; Yizhar et al., 2011), the low, medium, and high powers we used (1, 2.5, and 6.5 mW) lead to intensities 1 mm away (0.19, 0.5, and 1.25 mW/mm^2^) that (for medium and high powers) are within the range of ChR2 activation (Lin, 2011; Mattis et al., 2011). Second, we tested the light spread directly in urethane-anesthetized Vgat-ZI-ChR2 mice (see methods in Hormigo et al., 2016) by recording single-units in zona incerta while continuous pulses (0.5–1 s) of blue light were applied above the recording electrode. Application of light intensities estimated to reach zona incerta from PO thalamus when high power is used (1–2 mW/mm^2^) evoked robust firing (above baseline) in all the ChR2-expressing zona incerta cells tested (*n* = 7; Tukey, *p* < 0.0001, Cont vs spontaneous firing). In contrast, trains (1-m pulses at 40 Hz) were largely ineffective at these intensities; trains became highly effective as the blue light intensity increased. Thus, high-power blue light applied as continuous pulses in PO thalamus can directly excite ChR2-expressing cells in the underlying zona incerta, which suppresses avoidance responses (as shown in Fig. 4*A*). In conclusion, inhibition of PO thalamus cells does not suppress active avoidance, and excitation of zona incerta cells does not suppress active avoidance by inhibiting PO thalamus cells.

### Excitation of zona incerta GABAergic fibers in superior colliculus

Since inhibition of PO thalamus cells does not block active avoidance, we next tested the effect of exciting zona incerta GABAergic fibers in the superior colliculus or PPT on active avoidance.

**Figure 7.**
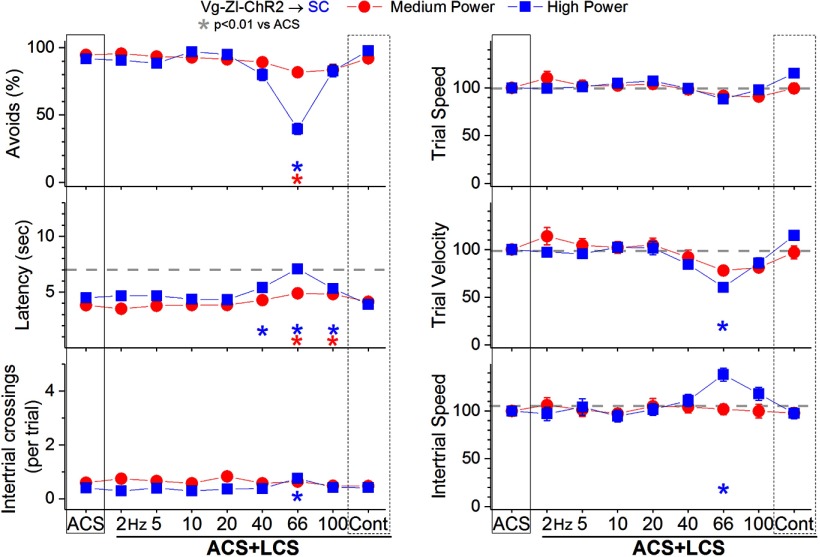
Effect of activating zona incerta GABAergic output fibers in superior colliculus on active avoidance responses. Effect of medium-power and high-power blue light applied in the superior colliculus on ACS+LCS trials for animals that express ChR2 in GABAergic fibers originating in the zona incerta (Vgat-ZI-ChR2→SC). The right panels show trial speed, trial velocity, and intertrial speed for the data in the left panels. The *x*-axis denotes blue light frequency trains of 1-ms pulses (Hz) or continuous pulses (Cont).

In ACS+LCS trials, excitation of zona incerta GABAergic fibers in the superior colliculus (incertocollicular fibers) with medium-power blue light (Fig. 7, red closed circles; 28 sessions in four mice) only marginally suppressed (∼10%) the percentage of avoidance responses (*p* < 0.0001 and *p* = 0.014; 66 and 100 Hz vs ACS), increased latency (*p* < 0.0001 and *p* < 0.0001; 66 and 100 Hz vs ACS), and suppressed trial velocity (*p* = 0.01; 66 Hz vs ACS) for blue light trains at 66–100 Hz compared with ACS trials. Increasing the blue light to a high power (Fig. 7, blue closed squares; 28 sessions in four mice) increased the suppressing effect but only for trains at around 66 Hz. Thus, high-power blue light suppressed the percentage avoidance responses (*p* < 0.0001; 66 Hz vs ACS), increased latency (*p* < 0.0001, *p* < 0.0001, and *p* = 0.005; 40, 66, and 100 Hz vs ACS) and suppressed trial velocity (*p* < 0.0001; 66 Hz vs ACS). The number of intertrial crossings (*p* < 0.0001; 66 Hz vs ACS) and intertrial speed (*p* < 0.0001; 66 Hz vs ACS) increased after ACS+ LCS trials in which avoidance responses had been blocked (i.e., 66 Hz). Therefore, excitation of zona incerta GABAergic fibers in the superior colliculus does not suppress signaled active avoidance responses, unless high-power blue light trains at around 66 Hz are used.

The effects on active avoidance of inhibiting superior colliculus cells by exciting GABAergic fibers originating in the zona incerta (incertotectal) resemble the effects of exciting GABAergic fibers originating in the SNr (nigrotectal; Hormigo et al., 2019). In both cases, activation of GABAergic fibers in superior colliculus within a narrow train frequency window (∼66 Hz) at high powers suppresses avoidance responses. However, it is not possible to ascribe this effect to the inhibition of superior colliculus cells because direct inhibition of superior colliculus cells (with eArchT3.0) does not suppress avoidance responses (Hormigo et al., 2019) and neither do lesions of the superior colliculus alone (Cohen and Castro-Alamancos, 2007). Thus, the very narrow suppressive effect of exciting incertotectal GABAergic fibers in superior colliculus (66-Hz train) that occurs only when high-power light is used must have a different explanation. Previously, it has been shown that the effects of exciting nigrotectal GABAergic fibers in superior colliculus on active avoidance could be explained by the antidromic excitation of nigrotectal cells that have collaterals that also project to the PPT in the midbrain (Hormigo et al., 2019). Indeed, zona incerta cells project through collaterals to several targets in the midbrain (Mitrofanis, 2005). Thus, we next tested the effect of exciting zona incerta GABAergic fibers in the PPT.

### Excitation of zona incerta GABAergic fibers in PPT blocks active avoidance

**Figure 8.**
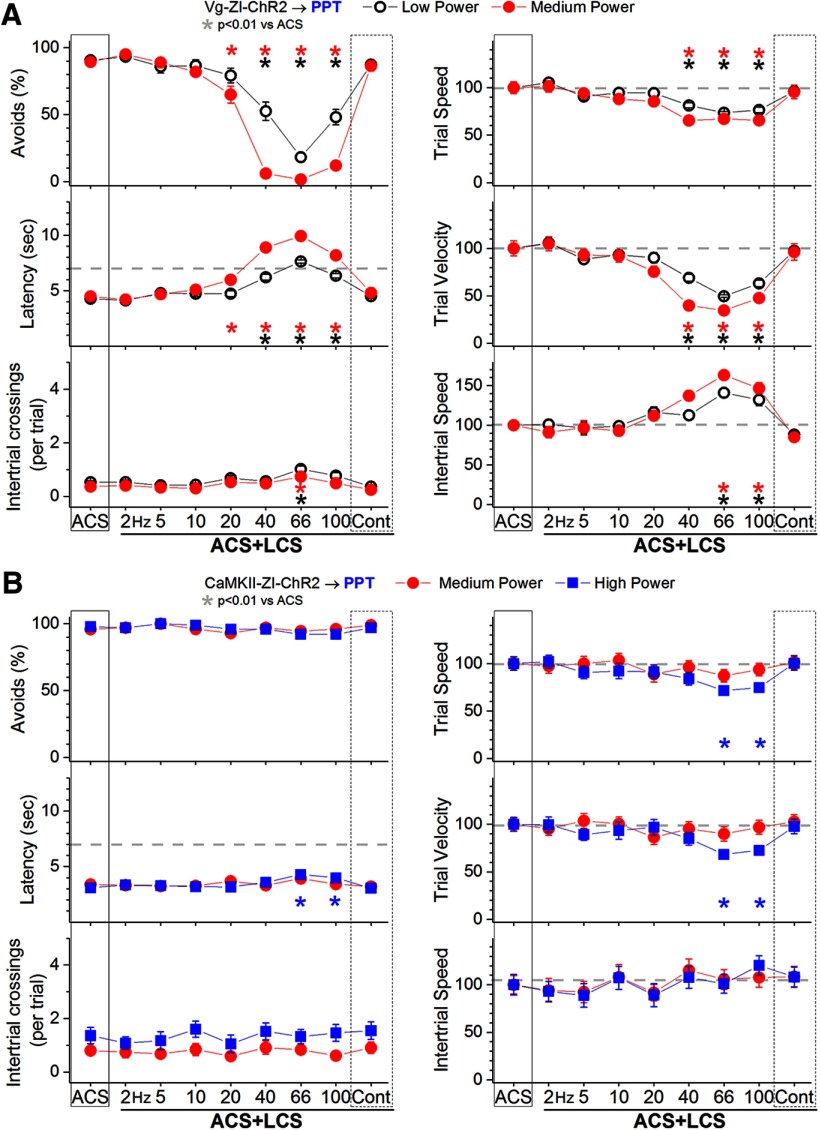
Effect of activating zona incerta GABAergic output fibers in PPT on active avoidance responses. ***A***, Effect of low-power and medium-power blue light applied in the PPT on ACS+LCS trials for animals that express ChR2 in GABAergic fibers originating in the zona incerta (Vgat-ZI-ChR2→PPT). The right panels show trial speed, trial velocity, and intertrial speed for the data in the left panels. The *x*-axis denotes blue light frequency trains of 1-ms pulses (Hz) or continuous pulses (Cont). ***B***, Effect of medium-power and high-power blue light applied in the PPT on ACS+LCS trials for animals that express ChR2 in CaMKII-expressing cells originating in the zona incerta (CaMKII-ZI-ChR2→PPT). The right panels show trial speed, trial velocity, and intertrial speed for the data in the left panels. The *x*-axis denotes blue light frequency trains of 1-ms pulses (Hz) or continuous pulses (Cont).

In ACS+LCS trials, excitation of zona incerta GABAergic fibers in the PPT (incertotegmental fibers) with low-power blue light at 40–100 Hz (Fig. 8*A*, black open circles; 27 sessions in four mice) strongly suppressed the percentage of avoidance responses (*p* < 0.0001, *p* < 0.0001, and *p* < 0.0001; 40, 66, and 100 Hz vs ACS), increased latency (*p* < 0.0001, *p* < 0.0001, and *p* < 0.0001; 40, 66, and 100 Hz vs ACS), and suppressed trial speed and trial velocity (*p* < 0.0001, *p* < 0.0001, and *p* < 0.0001; 40, 66, and 100 Hz vs ACS). Increasing the blue light to medium power (Fig. 8*A*, red closed circles; 21 sessions in four mice) increased the effect, but it was still centered on the same frequencies around 66 Hz. Thus, medium-power blue light trains suppressed the percentage of avoidance responses (*p* < 0.0001, *p* < 0.0001, *p* < 0.0001, and *p* < 0.0001; 20, 40, 66, and 100 Hz vs ACS), increased latency (*p* < 0.0001, *p* < 0.0001, *p* < 0.0001, and *p* < 0.0001; 20, 40, 66, and 100 Hz vs ACS), and suppressed trial speed and trial velocity (*p* < 0.0001, *p* < 0.0001, and *p* < 0.0001; 40, 66, and 100 Hz vs ACS). The number of intertrial crossings (*p* = 0.005; 66 Hz vs ACS) and intertrial speed (*p* < 0.0001 and *p* = 0.003; 66 and 100 Hz vs ACS) increased after ACS+LCS trials in which avoidance responses had been blocked (i.e., 66 Hz).

Thus, excitation of zona incerta GABAergic fibers in the PPT with low-power blue light trains at around 66 Hz strongly suppresses signaled active avoidance responses. These results are in close agreement with the effects of exciting nigrotegmental GABAergic fibers in PPT or directly inhibiting glutamatergic cells in PPT, which abolish signaled active avoidance (Hormigo et al., 2019). It is important to emphasize that the active avoidance suppression observed in PPT is robust for a broad range of optogenetic frequencies delivered at miniscule light intensities (low power). In contrast, the active avoidance suppression observed in superior colliculus (Fig. 7) only occurs for a narrow optogenetic stimulation frequency (66 Hz) and requires much higher (an order of magnitude higher) light intensities (high power).

The results indicate that excitation of GABAergic projections from zona incerta to PPT block signaled active avoidance. We next tested whether excitation of non-GABAergic (CaMKII-expressing) zona incerta cells projecting to PPT also affect avoidance. In a group of C57BL/6J mice (*n* = 4), we injected into the zona incerta an AAV with a CaMKII promoter to express ChR2 in CaMKII-expressing zona incerta cells (AAV5-CaMKIIa-hChR2(H134R)-EYFP; UNC Vector Core). These animals were implanted with bilateral optical fibers in the PPT (CaMKII-ZI-ChR2→PPT). Consistent with the well-known expression of CaMKII by thalamic cells (Wang et al., 2013), the AAV injections produced significant expression of ChR2 in thalamic cells overlying the zona incerta, but comparatively weak and sparse expression within zona incerta. Recordings from CaMKII-ZI-ChR2 slices revealed only weak excitatory field-potential responses in the PPT evoked by blue light (*n* = 8 slices from four mice; data not shown), indicating that this pathway is sparse.

In ACS+LCS trials, excitation of zona incerta CaMKII-expressing fibers in the PPT with medium-power or high-power blue light had no effect on the percentage of avoidance responses (*F*_(8,120)_ = 1.5, *p* = 0.14), response latency or the number of intertrial crossings (Fig. 8*B*, 20 sessions in four mice). Video tracking revealed a small suppression of trial speed and velocity but only for high-power trains at 66–100 Hz (*p* < 0.001 and *p* < 0.01; 66 and 100 Hz vs ACS). These results indicate that the control exerted by zona incerta over signaled active avoidance in PPT is selective for zona incerta GABAergic cells, and does not occur for CaMKII-expressing zona incerta cells.

In conclusion, excitation of zona incerta GABAergic cells suppresses signaled active avoidance responses primarily by inhibiting cells in PPT, not by inhibiting cells in PO thalamus or superior colliculus.

## Discussion

Inhibition of zona incerta GABAergic cells facilitates signaled active avoidance responses and drives them in the absence of an external signal. Conversely, excitation of zona incerta GABAergic cells, but not of NRT GABAergic cells, abolishes signaled active avoidance responses. Zona incerta GABAergic cells suppress signaled active avoidance primarily by inhibiting cells in the midbrain PPT, not by inhibiting cells in PO thalamus or superior colliculus. Moreover, zona incerta CaMKII-expressing cells that project to PPT do not suppress signaled active avoidance. Similar to the effects of modulating the GABAergic output of the basal ganglia through SNr (Hormigo et al., 2019), inhibiting zona incerta GABAergic cells drives avoidance responses, whereas exciting these cells suppresses avoidance responses.

### PO thalamus, superior colliculus, and NRT are not critical for active avoidance

Both the PO thalamus and superior colliculus are robustly inhibited by zona incerta GABAergic fibers, but neither of these target areas appear to be critical for signaled active avoidance. Indeed, direct optogenetic inhibition (with eArchT3.0) of PO thalamus cells (present study) or superior colliculus cells (Hormigo et al., 2019) has little effect on signaled active avoidance. Intriguingly, excitation of GABAergic afferent fibers in superior colliculus (selectively at 66 Hz), originating either in zona incerta (present study) or SNr (Hormigo et al., 2019), is capable of blocking signaled avoidance responses when light is delivered at high intensities. However, we have shown that this effect is due to the antidromic excitation of colliculus-projecting GABAergic cells that have fiber collaterals that also project to the PPT in the midbrain (Hormigo et al., 2019). Thus, when these GABAergic afferents are excited in superior colliculus, they also inhibit PPT. In addition, here, we also found that relatively high intensities of continuous blue light applied in PO thalamus, to excite GABAergic fibers, actually suppressed signaled active avoidance responses by directly exciting other ChR2-expressing cells in the underlying zona incerta (due to light spread). All these minutiae highlight the importance of testing different patterns and intensities of optogenetic stimulation, applying different optogenetic methods to inhibit target cells (synaptic inhibition by exciting GABAergic afferents, direct inhibition with Arch, etc.) and validating the approaches with electrophysiological recordings.

While superior colliculus cells do not appear to be critical for signaled active avoidance, superior colliculus cells fire during signaled active avoidance responses (Cohen and Castro-Alamancos, 2010b), and their excitation drives avoidance and escape responses (Shang et al., 2015; Wei et al., 2015; Hormigo et al., 2019). Thus, a main role of superior colliculus in signaled active avoidance appears to be in the detection and processing of the CS (Cohen and Castro-Alamancos, 2007, 2010a). Indeed, a wide variety of modulations of glutamatergic or GABAergic cells in the superior colliculus are effective conditioned signals to drive avoidance responses (Hormigo et al., 2019). Moreover, the superior colliculus has a well-known role in detecting sensory stimuli that require immediate action, such as in orienting behaviors (Wurtz and Hikosaka, 1986; Dean et al., 1989; Stein and Meredith, 1993; Hikosaka et al., 2000; Cohen et al., 2008; Felsen and Mainen, 2008; Gandhi and Katnani, 2011; Krauzlis et al., 2013). Thus, the superior colliculus, while not required for signaled active avoidance, seems to function as one of the redundant (parallel) sensory relays that can provide the PPT with CS-related signals necessary to drive avoidance responses.

The present results indicate that PO thalamus is not a critical part of the neural circuits required for the expression of signaled active avoidance. PO thalamus and its connections with zona incerta may serve other functions (Trageser and Keller, 2004; Lavallée et al., 2005; Trageser et al., 2006; Watson et al., 2015).

The results also revealed that excitation of NRT cells, which are well known to inhibit thalamic nuclei (Jones, 2002), does not suppress signaled active avoidance. This agrees with previous findings indicating that lesions of sensory thalamus alone do not block signaled active avoidance (Cohen and Castro-Alamancos, 2007, 2010a). The impetus to study NRT was to determine the selectivity of the zona incerta effects, since NRT cells are also GABAergic, inhibit large portions of the thalamus, are located adjacent to zona incerta, and do not project to the midbrain. The fact that excitation of NRT GABAergic cells does not suppress signaled active avoidance highlights the selectivity of the suppression of signaled active avoidance caused by exciting zona incerta GABAergic cells. Moreover, since NRT inhibits many thalamic nuclei, and this does not suppress signaled active avoidance, the suppression of active avoidance caused by excitation of zona incerta GABAergic cells must occur outside of the thalamus, such as in the midbrain.

### Converging GABAergic pathways control active avoidance in PPT

Inhibiting PPT cells by exciting zona incerta GABAergic fibers is highly effective at blocking avoidance responses, which further emphasizes the notion that the PPT is a critical junction for the expression of active avoidance responses. Indeed, inhibition of glutamatergic PPT cells is sufficient to block signaled active avoidance, while excitation of these PPT cells drives avoidance responses very effectively (Hormigo et al., 2019). Together, PPT and cuneiform nuclei form the MLR, an area known to regulate locomotion (Shik et al., 1966; Skinner and Garcia-Rill, 1984; Ryczko and Dubuc, 2013; Roseberry et al., 2016; Xiao et al., 2016; Caggiano et al., 2018). The PPT is composed of cholinergic, GABAergic, and glutamatergic neuronal subtypes (Wang and Morales, 2009; Mena-Segovia and Bolam, 2017), but it is the glutamatergic cells that seem to mediate avoidance responses (Hormigo et al., 2019). PPT GABAergic afferents originating within PPT, SNr or zona incerta are highly effective at suppressing signaled active avoidance responses. This regulation is very powerful since minute intensities of blue light applied in PPT to excite GABAergic afferents completely block signaled active avoidance, while preserving the ability of the animal to escape the US. Thus, both zona incerta and basal ganglia GABAergic outputs provide the PPT with parallel pathways to regulate signaled active avoidance responses. These pathways may share redundant roles in signaled active avoidance, or they may have specializations related to their overall functions. For example, SNr pathways to PPT may regulate avoidance responses by fine tuning speed and direction (velocity; Yin, 2014; Yttri and Dudman, 2016). Indeed, patterns of excessive activation of these GABAergic pathways, as may occur in Parkinson’s disease (Kravitz et al., 2010; Willard et al., 2019), will abnormally suppress signaled locomotor actions, leading to the well-known symptoms of this disorder. However, these channels surely do not exist to create pathology, and are likely engaged in regulating normal locomotor actions as demanded by behavioral contingencies. Indeed, these pathways receive extensive direct and indirect inputs from forebrain circuits, such as amygdala, frontal cortex and striatum, proposed to have significant roles in signaled active avoidance (Roozendaal et al., 1993; Choi et al., 2010; Lichtenberg et al., 2014; Bravo-Rivera et al., 2015; Ramirez et al., 2015). Forebrain circuits can control active avoidance by modulating SNr and zona incerta GABAergic cells. These channels provide direct links for higher order areas to access the basic circuits required for active avoidance expression.

### What is the role of zona incerta in active avoidance?

The results indicate that inhibition of zona incerta GABAergic cells drives active avoidance in the absence of any external sensory stimulus, but not in naive animals. Thus, inhibition of zona incerta GABAergic cells functions like a true CS that only begins to drive avoidance responses when it predicts the US. One possibility is that inhibition of zona incerta GABAergic cells disinhibits (excites) target neurons in sensory areas (e.g., superior colliculus, thalamus), which becomes conditioned just like the neural activity evoked by a natural sensory stimulus. Thus, by controlling the activity of targeted sensory nuclei, the zona incerta may have a role in regulating the effectiveness of the sensory CS, up to the point that it can replace it. On the other hand, excitation of zona incerta GABAergic cells blocks active avoidance by inhibiting cells in PPT. The extent to which zona incerta GABAergic cells are excited or inhibited during signaled active avoidance cannot be determined from the present study. This will require recording from these cells during signaled active avoidance.

## Synthesis

Reviewing Editor: Alexxai Kravitz, Washington University in St. Louis Decisions are customarily a result of the Reviewing Editor and the peer reviewers coming together and discussing their recommendations until a consensus is reached. When revisions are invited, a fact-based synthesis statement explaining their decision and outlining what is needed to prepare a revision will be listed below. The following reviewer(s) agreed to reveal their identity: Wei Shen.

The reviewers and I have come to an agreement that the following points should be addressed in a re-submission. These can largely be addressed through additions (or reductions in one case) to the text. However there is one control experiment that should be completed and will require a new group of opsin-expressing animals. This comment is presented first in this list:

1) The authors should address potential motor effects from their manipulations, and how they may contribute to their findings on avoidance. While new control groups are not necessary for all manipulations of the paper, one new control experiment should be completed for the manipulation that tested their primary conclusion. The authors should generate new naive animals that express ChR2 in gabaergic neurons of the ZI, and see if 1) stimulation of ZI gabaergic neurons decreases movement and/or causes postural changes in the absence of any task or prior training; and 2) stimulation of the ZI gaba neurons in naive, untrained animals, can produce the appearance of altering avoidance behavior if scored identically to those trained on the task, due to motor effects during stimulation.

2) The authors should discuss the frequency dependence of their effects, and explain why they think they didn’t see effects until very high frequencies of stimulation.

3) The authors should include a solid section in the Discussion addressing how they think that the ZI-PPT connection specifically contributes to goal-directed avoidance behavior. Is there a specific role for the ZI-PPT projection beyond what can be accounted for by altering PPT activity alone?

4) The aspects of the paper dealing with PO thalamus and superior colliculus should be greatly shortened or removed, for the reasons brought up by Reviewer 2 below.

5) Both reviewers also provided some more minor comments, which should all be addressed.

Full reviewer comments below:

****************************************************

Reviewer 1

****************************************************

The manuscript used a different assay (active avoidance) to analyze the defense behavior, which is in supplement to the sound induced avoidance behavior used by Chou, X. L. et al. Nat Commun 9, 1151, and highlighted the ZI as a center to regulate various defense behaviors. It projected to the PPT to exert this role, which they identified a new projection from the ZI other than the ZI-PAG described by Chou, X. L. et al. Nat Commun 9, 1151. Here are some issues they need to address.

1. They forgot to cite an important paper for ZI function in prey hunting and motivation (Zhao, Z. D. et al. Nat Neurosci 22, 921-932). In Zhao et al and Chou et al, they clearly defined the bregma sites for optogenetic stimulation. As the ZI may have functional segregation from anterior to posterior. The authors should clarify by showing histology or cFos.

2. It is weird that the authors had to use very high frequency to see an effect (Fig. 2), please explain.

3. The author inhibited the PO to check whether they are required for avoidance, they should do the same for the PPT also.

4. Had the authors checked the ZI-PAG reported by Zhao et al and Chou et al. It might be interesting to see whether it is also required in this active avoidance behavior.

****************************************************

Reviewer 2

****************************************************

Authors (Required) The current manuscript investigates the contribution of the zona incerta to the expression of signaled active avoidance. The experiments and the analysis are well performed and the work clearly shows that manipulation of the zona incerta influences the avoidance behavior. At the same time, the way in which these manipulations may influence the behavior is not really addressed in the Discussion. I would like the authors to give a better idea of where they think that the zona incerta sits in the complex network that is producing this behavior. As part of this Discussions they should also better compare the role of the zona incerta with respect to that of other structures that have been studied in the same context.

Major comments

As indicated in the general comments, it is not clear from these experiments how the zona incerta influences avoidance. The manuscript discusses the fact that the zona incerta can “facilitate” avoidance behavior and that the pedunculopntine tegmentum (PPT) is responsible for “expressing” the behavior (ln 625). However, the most important finding is that the zona incerta can “drive” the behavior. This is based on the finding that optical stimulation of the zona incerta in the absence of any conditioning stimulus is able to produce avoidance behavior. Indeed, at the highest strengths of stimulation used, the avoidance is as efficient as the avoidance produced by the conditioning stimulus and is superior to the avoidance observed in trials with no stimulation and no conditioning stimulus (No-CS). While this is certainly an interesting finding, I am not totally convinced that this is proof that the zona incerta is specifically driving the behavior. Isn’t it possible that this is the result of increased (or decreased) locomotor behavior on the part of the mouse that, with nowhere else to go, moves into the other part of the shuttle apparatus? After all, activation of the MLR can initiate various types of locomotion as acknowledged in the list of references. One imagines that if one activates a locomotor region that this will lead to the mouse moving and following the only path available to it. The No-CS condition does not control for this non-specific effect caused by increased locomotor activity.

As some support for their position the authors note that inactivating the PPT blocks signaled avoidance but not the response to the unconditioned stimulus (ln 635). However, this seems rather to be comparing apples and oranges. One is a reaction to a painful stimulus and the other a conditioned response. I do not see that this statement can demonstrate specificity for the conditioned response. A more suitable control might be to use the optogenetic stimulation on naïve mice to determine if they moved no more frequently from one side of the apparatus to the other than mice with no stimulation. Certainly, there needs to be a better discussion of whether this protocol is sufficient to show that activation of the MLR is sufficient to specifically produce such a complex goal-directed behavior.

In this same line, it is not clear from the manuscript, whether the authors believe that the zona incerta, and the SNpr each have specific roles to play in the avoidance behavior. Given the information provided in the Introduction and in Hormingo et al. 2019, it seems the bottom line is that the PPT is ultimately implicated in the modification of the avoidance behavior (with the caveats given above). As such, wouldn’t one expect that any structure (including the zona incerta) that modifies PPT activity would also modify avoidance behavior? If the authors believe that the zona incerta brings some unique contribution to the behavior, beyond simply modifying PPT activity, then this should be addressed in the Discussion, and better introduced.

The rationale and necessity of performing and detailing the experiments involving manipulation of the PO thalamus and the superior colliculus is not clear. For example, it appears that the zona incerta sends collaterals to both structures so that stimulation of zona incerta fibers in one structure is likely to activate other target structures via these collaterals, as finally noted on ln 510. In addition, in the experiments examining the contribution of the PO thalamus, we learn that that the stimulus is very likely directly activating zona incerta cells and control experiments, indeed, show that the stimuli used modulated the activity of zona incerta cells. So, why present all of the results from stimulating PO thalamus if the methodology was inappropriate to selectively activate the PO? This section needs to be drastically shortened to simply state that no effects were observed from the PO thalamus unless the stimulus was increased to levels that caused contamination. Lastly, a similar argument concerns the section on the superior colliculus. No rationale for thinking that the superior colliculus is involved in this behavior is provided (in this manuscript) and later in the section we find that direct inhibition of the superior colliculus (tested in other experiments) does not suppress avoidance responses. While there is useful information in these sections, it does not deserve 4 pages and should be shortened by at least 50%. The section discussing these results (ln 573 and following) could also be simplified and shortened.

There is no information on the effect of the stimulation on the general posture and locomotor activity of the mouse. For example, Takakusaki, has shown how stimulation of the substantia nigra pars reticulata (SNpr) can modify postural support and how stimulation of different regions of the MLR can either increase or decrease posture. The work of Caggiano et al. further shows that activation of different populations of neurons in the MLR can produce different types of locomotion. One would think that such changes would influence the avoidance behavior but no information or discussion of these points is provided (at least in this manuscript).

Minor Comments

The first section of the Results effectively repeats the information on page 10.

The rationale for the experiments involving the thalamic reticular nucleus (NRT) needs to be better explained. Currently (ln 366 and subsequent paragraph) there seems to be little reason for believing that the NRT may have a similar effect to the zona incerta. It is only at the end of this section that we learn that the zona incerta also has strong projections to the thalamus and that the authors are determining if activation of the thalamus might be responsible for the effects that they observe. This latter rationale would be better placed at the beginning of the section.

Specific comments

Ln 35 None of these references are in the reference list.

Ln 39 “crossing the street in response to the appropriate light” would hardly seem to be an “avoidance behavior”.

Ln52 The rationale for the experiments provided in the first paragraph of the Introduction seems more interesting than the one presented here. Similarly, the statements in the following sentences hardly seem to make a strong case for an implication of the zona incerta in the control of signaled avoidance.

Ln396 Please provide a reference for these projections.

Ln 613 This rationale for studying the NRT also does not seem very useful.

Ln 628 Which reference includes the mesencephalic reticular nucleus as part of the MLR?
